# Adherence of staphylococcus aureus to catheter tubing inhibition by quaternary ammonium compounds

**DOI:** 10.11604/pamj.2016.25.50.8134

**Published:** 2016-09-29

**Authors:** Jean-Marie Liesse Iyamba, Daniel Tassa Okombe, Francis Nsimba Zakanda, Trésor Kimbeni Malongo, Joseph Welo Unya, Cyprien Mbundu Lukukula, Ntondo za Balega Takaisi Kikuni

**Affiliations:** 1 Laboratory of Experimental and Pharmaceutical Microbiology, Faculty of Pharmaceutical Sciences, University of Kinshasa, Kinshasa, Democratic Republic of Congo; 2 Laboratory of Biochemistry, Faculty of Medicine, University of Kinshasa, Democratic Republic of Congo; 3 Laboratory of Instrumental and Bioelectrochemical analysis, Faculty of Pharmaceutical Sciences, University of Kinshasa, Kinshasa, Democratic Republic of Congo

**Keywords:** Staphylococcus aureus, quaternary ammonium, betaine ester, catheter, biofilm

## Abstract

**Introduction:**

*S. aureus* is a Gram positive bacterium which is responsible for a wide range of infections. This pathogen has also the ability to adhere to biotic or abiotic surface such as central venous catheter (CVC) and to produce a biofilm. The aim of this study was to evaluate the effect of hexadecyltrimethyl ammonium bromide (HTAB) and Hexadecylbetainate chloride (HBC) on *Staphylococcus aureus* adherence to the catheter tubing and on bacteria growth.

**Methods:**

Broth microdilution method was used to determine the Minimal Inhibitory Concentration (MIC). The detection of slime production was done by Congo Red Agar method, and the adherence of bacteria to the catheter tubing was evaluated by the enumeration of bacteria on plate counts.

**Results:**

The results of this study showed that the MICs of HTAB were ranged from 0.125 to 0.5 µg/mL, and those of HBC fluctuated between 2 to 8 µg/mL. HTAB and HBC inhibited bacteria adhesion on the surface of the catheter tubing.

**Conclusion:**

This study showed that HTAB and HBC can prevent the adherence of *S. aureus* strains to the surface of catheter tubing, suggesting that they could be used to prevent the risk of catheter related bloodstream infections.

## Introduction

The Gram-positive bacterium *Staphylococcus aureus* is considered to be a virulent pathogen responsible for nosocomial and hospital acquired infections [1]. *S. aureus* possesses numerous virulence factors which facilitate its ability to infect a wide variety of body tissues [2]. *S. aureus* is an important cause of purulent soft-skin infections, bacteremia, and deep-seated infections including osteomyelitis, septic arthritis, endocarditis, and pneumonia [3]. In addition, numerous methicillin resistant *S. aureus* strains (MRSA) are now multi-drug resistant [4, 5]. *S. aureus* has also the ability to adhere on a biotic or abiotic surface and to form a biofilm which is defined as aggregated, microbial cells surrounded by a polymeric self-produced matrix, which may contain host components [6]. The ability to adhere and to promote formation of a biofilm on a tissue or on the plastic surface of indwelling medical devices is an important feature of the pathogenicity of *S. aureus* foreign body infections. Biofilm-associated bacteria are generally resistant to antibiotics, and host defenses [7, 8]. Bacterial adhesion on implants remains the first and key step in the development of chronic body associated infections [9]. Finding strategies to minimize *S. aureus* adhesion on an abiotic surface may contribute to reduce such infections. Previous studies have demonstrated that the inhibition of microbial adhesion to the device surfaces may be obtained by the coating of the catheter with hydrophilic polymers, antimicrobial agents, benzalkonium chloride, chlorhexidine/silver sulfadiazine, silver, trimethylsilane, or furanones [10]. Catheter lock solutions containing antibiotics or chelator agents are also used to prevent bacteria adhesion and the formation of biofilm on a medical device [11, 12]. Among antiseptics, quaternary ammonium compounds and esters of betaine are used against a broad range of microorganisms [13, 14]. These compounds contain at least one long hydrophobic alkyl chain attached to a positively charged central nitrogen atom, which is the functional part of the molecule. The positive charge of nitrogen interacts with the negatively charged cell wall of bacteria, which disrupts the normal functions of the membrane [13]. Literature data demonstrated that antimicrobial activity of quaternary ammonium salts has been evaluated on planktonic bacteria and on biofilm formation [13–17]. To date, there has been no report on the effect of the newly synthetized betaine ester, hexadecylbetainate chloride, against *S. aureus* planktonic cultures and the initial stage of biofilm formation. The aim of this study was to assess the effect of hexadecyltrimethyl ammonium bromide in comparison with hexadecylbetainate chloride in vitro on the adhesion of *S. aureus* to the surface of catheter tubing and against planktonic cultures.

## Methods

### Compounds investigated

Hexadecyltrimethyl ammonium bromide (HTAB) was purchased from Sigma Aldrich (Germany). Hexadecylbetainate chloride (HBC) was prepared as described previously [18] (Table 1). Stock solutions (100 µg/mL) of the HTAB and HBC were prepared in sterile phosphate-buffer solution PBS (pH 7.2). Tested concentrations ranged from 0.125 to 64 µg/mL for both compounds.

**Table 1:** Chemical structures of compounds

Compounds	Chemical structures
Hexadecylbetainate chloride (HBC)	Cl^-^ ^+^N(CH_3_)_3_-CH_2_COOCH_2_(CH_2_)_12_CH_2_CH_3_
Hexadecyltrimethyl ammonium bromide (HTAB)	Br ^-^ ^+^N(CH_3_)_3_-CH_2_CH_2_(CH_2_)_12_CH_2_CH_3_

### Bacterial strains

The 12 clinical strains used in this study were collected for diagnostic purposes by the laboratory of bacteriology of the Biamba Marie Mutombo Hospital and were from different clinical specimens (Table 2). The two slime producers reference strains of S. aureus (the methicillin sensitive *S. aureus* (MSSA) ATCC-25923 and the methicillin resistant *S. aureus* (MRSA) ATCC-33591 strains) tested in this study were obtained from the American Type Culture Collection (Manassas, VA, USA). Bacteria were grown and isolated from clinical specimens in Mannitol Salt Agar (Liofilchem, Abruzzi, Italy). The identification of S. aureus was performed by latex agglutination test (Pastorex Staph-Plus, BioRad, Marnes-la-Coquette, France). The sensitivity of *S. aureus* to methicillin was examined with the diffusion method on Mueller Hinton Agar with 4% NaCl [19]. Bacterial suspensions equivalent to 0.5 MacFarland (108 colony-forming units (CFU)/mL) standards were used.

**Table 2:** Origin of the strains

Strains	Origin
**MRSA**	
281/CVV	Central venous catheter
286/ FV	Vaginal smear
291/CVV	Central venous catheter
292/P	Wound
ATCC33591	Reference
**MSSA**	
10/LP	Prostatic fluid
15/U	Urine
18/CVV	Central venous catheter
278/S	Blood
279/FV	Vaginal smear
280/LP	Prostatic fluid
250/FV	Vaginal smear
287/CVV	Central venous catheter
ATCC 25923	Reference

**CVV**: central venous catheter; **P**: wound; **FV**: vaginal smear; **U**: urine; **S**: blood; **LP**: prostatic fluid; **ATCC**: American Type Culture Collection

### Activity of HTAB and HBC on planktonic cultures

The activity of HTAB and HBC was tested on different *S. aureus* clinical and reference strains using the two-fold dilution method according to the Clinical and Laboratory Standard Institute (CLSI) guidelines [20]. Minimal inhibitory concentrations (MICs) were determined in 96-well microplates (Greiner BioOne). A suspension (108 CFU /mL) of the bacteria was added to the wells, and the microplates were incubated in aerobic conditions at 37°C for 18 hours. The MIC was defined as the lowest concentration of compound in which there was no visible growth after overnight incubation. For minimal bactericidal concentration (MBC) determination, 10 µL from the wells with no visible growth were subcultured in TSA plates and the agar plates were incubated overnight at 37°C. Growth results were then recorded.

### Detection of slime production

The slime production was carried out using the Congo Red Agar (CRA) method as described previously [21]. Briefly, *S. aureus* strains inoculated into Tryptone Soya Agar (TSA) (Difco, BD Franklin Lakes, NJ, USA) supplemented with 2% of glucose or 4% NaCl and 0.8% of Congo red, were incubated for 18 hours at 37°C under aerobic conditions and followed by incubation overnight at room temperature. Slime production was assessed on the basis of the color of staphylococcal colonies on CRA. Slime- producing strains give black colonies, whereas slime-negative strains develop red colonies. Colonies color on CRA was interpreted as follows: Very black and black colonies were considered to be normal slime-producing strains, whereas, very red and red colonies were classified as non- slime-producing strains [22].

### Effect of HTAB and HBC on the adherence of *S. aureus* to catheter tubing

The effect of the two compounds on adherence was studied as follows [23]: sterile fragments of a catheter tube (2 cm long, 3 mm inner diameter) were incubated for 18 hours at 37°C in the presence of a bacterial suspension (108 CFU/mL) into test tubes (in TSB glucose) as the control, or in the presence of the same bacterial suspension plus tested compounds. The concentration tested was 4 µg/mL which is within the range quoted for commercial quaternary ammonium compounds [24]. After incubation, the catheter tubes were removed in a sterile way, rinsed twice with sterile water before adding 1 mL PBS (pH 7.2). The cells were detached from the tubing by incubation in an ultrasound bath at 25°C for 5 min. After serial dilutions of the bacterial suspension with PBS, the bacteria were plated on Petri dishes containing 15 mL TSA medium and the Petri dishes were incubated at 37°C for 48 hours before counting the colonies. Dishes with less than 50 colonies or with more than 30 colonies were not counted. The assay was performed in triplicate and the results are represented as the mean CFU count.

### Statistical analysis

The statistical analysis was performed with GraphPad Prism 4.0. Results were analyzed using a one way ANOVA followed by Kruskall Wallis test. ***P < 0.005, **P < 0.01, *P < 0.05.

## Results

### Inhibitory activity of HTAB and HBC on planktonic cultures

The MIC of HTAB and MBC on planktonic culture of *S. aureus* strains are shown in **Table 3**.

**Table 3:** Susceptibility of *S. aureus* strains to HTAB and HBC

Strains	HTAB		HBC	
	MIC (µg/mL)	MBC (µg/mL)	MIC (µg/mL)	MBC(µg/mL)
**MRSA**				
281/CVV	0.25	4	16	64
286/ FV	0.25	1	4	16
291/U	2	8	8	32
292/CVV	0.25	1	16	32
ATCC33591	0.5	4	4	16
**MSSA**				
10/LP	0.5	4	8	32
15/U	0.5	4	8	˃ 64
18/CVV	0.5	4	8	32
278/S	0.5	4	8	˃ 64
279/FV	0.125	2	8	16
280/LP	0.25	4	16	64
250/FV	0.125	2	8	˃ 64
287/CVV	0.125	8	4	32
ATCC 25923	0.125	4	4	16

**CVV**: central venous catheter; **P**: wound; **FV**: vaginal smear; **U**: urine; **S**: blood; **LP**: prostatic fluid; **ATCC**: American Type Culture Collection

The studied strains were similarly susceptible to HTAB with MICs ranged from 0.125 to 0.5 µg/mL, whereas, the MICs of HBC were higher (from 4 to 16 µg/mL) against the majority of strains. In general, the MRSA and MSSA strains were more sensitive to HTAB (MIC [µg/mL] = 0. 43 ± 0.18) than to HBC (MIC (µg/mL) = 8.57 ± 3.27). The MBCs of HTAB varied from 2 to 8 µg/mL against the majority of MRSA and MSSA strains, except for MRSA clinical strains 286/FV and 292/CVV which were equal to 1 µg/mL. In contrast High MBCs of HBC (= 16mg/mL) were observed against all studied strains of S. aureus.

### Detection of slime production

To investigate the ability of *S. aureus* strains to produce a biofilm, the environmental regulation of biofilm development by NaCl and glucose on aerobic condition was assessed in MRSA and MSSA strains using CRA plates. The results presented in Table 4 show that in the presence of NaCl, only the MRSA clinical strain 292/CVV was slime producer. Whereas in MSSA clinical strains, 4 of 8 strains were slime producers. In the other hand, we observed that the addition of 2% glucose to CRA, the MRSA clinical strains 281/CVV and 292/CVV became slime producers. Interestingly, all the MSSA clinical strains were slime producers, in exception of the 2 strains 10/LP and 15/U which were also non-slime producers in the presence of NaCl.

**Table 4:** Slime producing *S. aureus* strains

	2% Glucose			4 % NaCl		
	Phenotype of strains (CRA)			Production of slime	Phenotype of strains (CRA)			Production of slime
	Aerobic (37°C/24 h)	Room temperature		Aerobic (37°C/24 h)	Room temperature	
Strains	**MRSA**			**MRSA**		
281/CVC	Very black	Very black	Producer	Red	Red	Non- producer
286/ FV	Red	Red	Non- producer	Red	Red	Non- producer
291/SU	Red	Red	Non- producer	Red	Red	Non- producer
292/CVC	Black	Black	Producer	Black	Black	Producer
ATCC33591	Black	Very black	Producer	Black	Black	Producer
	**MSSA**			**MSSA**		
10/LP	Red	Red	Non- producer	Red	Red	Non- producer
15/U	Red	Red	Non-Producer	Red	Red	Non- producer
18/CVC	Very black	Very black	Producer	Red	Red	Non- producer
278/S	Very black	Very black	Producer	Very black	Very black	Producer
279/FV	Black	Very black	Producer	Red	Red	Non- producer
280/LP	Black	Very black	Producer	Black	Very black	Producer
250/FV	Black	Very black	Producer	Black	Very black	Producer
287/CVC	Black	Very black	Producer	Black	Very black	Producer
ATCC 25923	Black	Very black	Producer	Black	Very black	Producer

**CVV**: central venous catheter; **P**: wound; **FV**: vaginal smear; **U**: urine; **S**: blood; **LP**: prostatic fluid; **ATCC**: American Type Culture Collection

### Effect of HTAB and HBC on the adherence of *S. aureus* to catheter tubing

**Figure 1:**
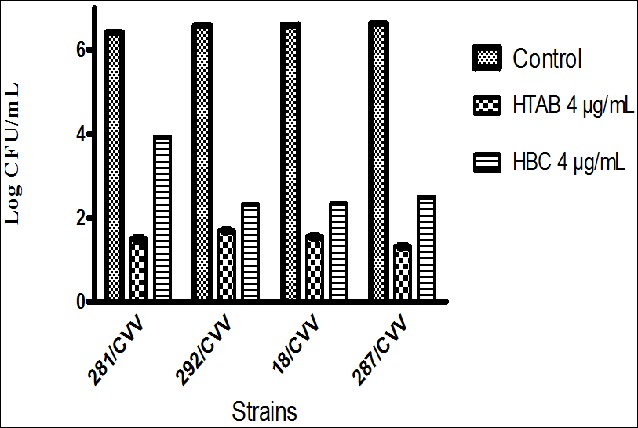
Adhesion of *S. aureus* strains to a catheter tubing

The effect of HTAB and HBC on the adherence of bacteria to fragments of a catheter tube was evaluated especially in slime producer strains of MRSA (281/CVV and 292/CVV) and MSSA (18/CVV and 287/CVV) isolated from CVV infections (Figure 1). For this purpose, we quantified the adherence of bacteria to a small piece of catheter tubing after incubation of 24 hours in the absence or in the presence of tested products as described in the protocol. The results obtained demonstrated that, at a concentration of 4 µg/mL, HTAB and HBC inhibited the adhesion of *S. aureus* CVV strains to the surface of catheter tubing in comparison with the control (P < 0.005). The adhesion of *S. aureus* clinical strain 287/CVV to catheter tubing was more affected by HTAB. In general, the effect of HTAB was slightly more effective than this of HBC (P < 0.05).

## Discussion

In vitro biofiofilm formation was performed in order to investigate whether 4% NaCl and 2% glucose could influence the capacity of MRSA and MSSA to form a biofilm on CRA plates. This study revealed that NaCl induced biofilm formation in 50% of MSSA clinical strains in aerobic conditions. Our results are consistent with those obtained by O’Neil et al, [25] who demonstrated the activation of biofilm formation by MSSA strains in BHI medium supplemented with 4% NaCl. Staphylococcal biofilm matrix contains polysaccharides, proteins, and extracellular DNA (eDNA) [26]. In spite of the fact that we have not investigated matrix composition, literature data showed that NaCl is an inducer of ica operon [27]. In the present study we also observed a great enhancement in the production of biofilm production by MRSA and MSSA strains in the presence of 2% glucose [25]. Media supplemented with glucose are used by many laboratories to induce staphylococcal biofilm formation in MSSA or MRSA strains [25, 26]. Staphylococcal biofilm formation plays a significant role in antibiotic resistance and contributes to the acquisition of severe acute or persistence of chronic infections, and hospital- acquired infections. In this work we investigated the effect of HTAB and HBC in bacteria growth and prevention of biofilm formation. The susceptibility testing using the broth microdilution method (MIC) indicated that HTAB and HBC were able to inhibit bacteria growth and to kill bacteria. Our results were consistent with those obtained in other previous studies [15, 14]. HTAB MICs values were lower or within the MIC range quoted for commercial quaternary ammonium compounds, 0.5 to 5.0 µg/mL [24] contrary to those of HBC (MIC from 2 to 8 µg/mL). Many quaternary ammonium salts are used as germicidal and disinfecting agents since they adsorb on the negatively charged bacterium surface, thereby they reduce the surface tension and perturb the function of cell membrane [28]. The two compounds tested possess 16 carbon atoms in their alkyl chains (Table 1), but they have different chemical structures, which could explain the difference in their respective antimicrobial activity. HTAB has a higher hydrophobic character than HBC due to the presence of the ester bond which increases its polarity. As *S. aureus* surface is very hydrophobic [29], the interaction with HTAB could be stronger enough to cause the disruption of the bacteria membranes. The HBC high MIC values observed is probably due to its weak interaction with the hydrophobic surface of *S. aureus* strains. The effect of HTAB and HBC were evaluated in the adherence of *S. aureus* on catheter tubing. The results from this study showed that the two compounds significantly inhibited bacterial adhesion on catheter tubing, although the inhibitory effect varied among the different strains. Better inhibition was observed at 4 µg/mL. The tested compounds have no effect on a preformed biofilm (data not shown). Comparing the effect of the 2 compounds on adhesion, we observed that at the same concentration, HTAB was slightly more effective than HBC. But any compound inhibited completely the adhesion of bacteria. It was observed that quaternary bis ammonium salt and cetylpyridinium bromide inhibited biofilm formation by S. epidermidis, but neither of the two compounds caused the eradication of staphylococcal biofilm [17]. According to the results obtained by these authors, the effect of HTAB and HBC in the initial stage of biofilm formation would probably due to the inhibition of bacteria growth.

## Conclusion

We have investigated the effect of HTAB and HBC on the initial stage of staphylococcal biofilm formation with the aim to search products which should be used as catheter lock solutions. Our results showed that HTAB and HBC at 4µg/ml can prevent the adhesion of bacteria in the surface of catheter tubing, suggesting that they could reduce or prevent the risk of catheter related bloodstream infections.

### What is known about this topic

- *S. aureus* is the most important pathogen responsible of a wide range of infections, and possesses the ability to adhere to medical devices and to form a biofilm;
- *S. aureus* biofilm associated infections are difficult to treat clinically because the bacteria within the biofilm are protected from phagocytosis, antibiotics and disinfectants;
- Biological evaluation of quaternary ammonium compounds against bacteria biofilm by staining method in 96-well tissue culture plates.

### What this study adds

- Biological evaluation HTAB and HBC in the earlier stage of biofilm formation using the catheter tubing model;
- HTAB and HBC affected in vitro the initial adherence of S. aureus strains onto catheter tubing;
- Our results established the effectiveness of lock solutions with HTAB and HBC to prevent the formation *in vitro* of biofilms by *S. aureus*.
